# On interpretation of the effects of noise on cognitive performance: the fallacy of confusing the definition of an effect with the explanation of that effect

**DOI:** 10.3389/fpsyg.2015.00754

**Published:** 2015-06-02

**Authors:** Patrik Sörqvist

**Affiliations:** Department of Building, Energy and Environmental Engineering, University of GävleGävle, Sweden

**Keywords:** interpretation, explanation, definition, circular argument, cognitive noise effects, noise, irrelevant sound effect

The effects of noise on human beings are of interest to specialists across many academic disciplines including medicine, economics, and psychology (Basner et al., 2015). In cognitive psychology, people's susceptibility to distraction by background sound/noise is often used as an instrument to understand the nature of selective attention and short-term memory, and many theories have been proposed to explain why people tend to perform better in silence compared with when there is noise in the background (Hughes, 2014). For example, one explanation of noise effects that has been offered is that noise captures attention. On this view, the reason why performance on a visual task (e.g., proofreading) is impaired by background noise, is that the locus of attention is diverted away from the target information (e.g., the visual text material) and is instead reallocated to the sound, causing interruption to the task (Bell et al., 2012). A competing explanation is that similar processes partake in the involuntary analysis of the background noise and the voluntary elaboration of the task material, causing a conflict and performance decrements (Macken, 2014). A third explanation, offered in the context of noise effects on complex cognition, such as that on reading comprehension or word processed writing, is that noise impairs subcomponent abilities (e.g., working memory) that is assumed to underpin the complex behavior (Jahncke et al., 2013).

In previous papers, I have tried to systematically discuss conceptual difficulties and lack of clarity in the cognitive noise literature. The intention has been, specifically, to clarify issues concerning explanation and interpretation of noise effects. In one paper, I discussed the conceptual problems associated with the attempt to pinpoint the cognitive structure (or memory system) that is impaired by background noise (Sörqvist, 2014). And in another paper, I discussed the problems associated with the idea that the effects of noise on complex cognitive behavior (e.g., reading comprehension) can be explained as an effect of noise on a sub-component of that complex behavior (Sörqvist, 2015). Here, in the current paper, I will address another conceptual issue: That we sometime confuse the definition of an effect with the explanation of that effect.

## Definition vs. explanation

In experimental psychology, an effect is the difference between two (or more) conditions. On this view, an effect of noise is the difference between a noise condition and a control condition (typically a silent condition). The well-known and extensively studied *irrelevant sound effect*, for example, is hence the difference in performance on a serial short-term memory task between a sound condition and a control condition. The *definition* of the effect *is* the difference on the dependent variable between two (or more) levels on the independent variable. An *explanation* of an effect, in turn, is a theoretical assumption as to why the difference between the conditions arises. It is very important to keep the difference between *definition* and *explanation* in mind, in particular when thinking about, and writing papers on, cognitive noise effects. When they are not kept apart, an explanation can easily become embedded into the definition, and that leads to conceptual difficulties; difficulties that shroud the true meaning of the effects, make theory testing troublesome and can potentially constrain scientific progress.

I will address one concrete example from the literature to make my point clear (i.e., that definition and explanation must be held apart, otherwise theory testing is compromised). When participants view a sequence of visually-presented items (e.g., k l m v r q c) and later attempt to recall those items in order of presentation, they perform better when the to-be-recalled items are presented together with a task-irrelevant sound stream with a single, repetitive sound element (e.g., d d d d d d d), in comparison with a condition wherein the to-be-recalled items are presented together with a sound stream with a deviant embedded (e.g., d d d K d d d). This difference in performance between a “steady state sound condition” and a “deviant sound condition” is (the definition of) the *deviation effect*. A potential explanation of this effect (i.e., a theoretical assumption as to why the difference between the two conditions arises) is that the auditory deviant (the “K” in the example above) captures attention, drawing attention away from the to-be-recalled material, and thereby causing a performance decrement.

It is not unusual that definition and explanation of effects—like the deviation effect—are confused. For instance, the definition of the deviation effect is sometimes offered as this: “An auditory deviation effect occurs when attention is drawn away from the primary task by an irrelevant but salient auditory deviant” (Meinhardt-Injac et al., 2015, p.217). In cases such as this, when the *explanation* is embedded in the *definition*, the definition pre-specifies the explanation. On this view, the reason why the deviation effect arises is because attention is drawn away from the primary task by the salient sound. Other potential explanations cannot be offered, since the explanation is a built-in part of the definition. If, for example, interference between similar processes is offered as an explanation for a noise effect (e.g., as an explanation to why a difference in recall performance have occurred between a “steady state sound condition” and a “deviant sound condition”), that noise effect could not be the deviation effect, because the deviation effect occurs when attention is captured, nothing else. Such a view of the deviation effect leads ultimately to circular reasoning.

## Circularity

“Circularity” (or circular argumentation) is a tricky concept. The following (hypothetical) conversation between two researchers will serve as a clarifying example of circular argumentation:

- Individual differences in working memory capacity reflect individual differences in attention abilities.
- How do you know?
- Well. Working memory capacity is related to performance on the Stroop task, and the Stroop task is a measure of attention abilities.
- How do you know that Stoop measures attention abilities?
- Because performance on the Stoop task depends on individual differences in working memory capacity, and, as we know, individual differences in working memory capacity reflect individual differences in attention abilities.

This is an example of a circular argument, because there is no independent evidence in favor of the basic premise—that the Stroop task measures attention abilities—and therefore the conclusion is self-reinforcing. Many influential theories in cognitive psychology have been challenged for their circularity—such as Craik and Lockhart's levels-of-processing theory (Baddeley, 1978), Lavie's load theory (Benoni and Tsal, 2013) and Baddeley's phonological loop model (Jones et al., 2007)—but they are still very influential, successful, and promote a substantial amount of good research. The reason why circularity is acceptable to some degree is that circular theories may still propose the best explanation from a probability viewpoint (Hahn and Oaksford, 2007).

Consider the following example as an illustration of a circular argument from the noise literature: We know that serial recall of a list of visually-presented items (e.g., “Monday, Saturday, Thursday, Tuesday, … ”) is more impaired when the visual list is studied in the presence of a changing-state sound sequence (e.g., “k l m v r q c”) than in the presence of a steady-state sound sequence (e.g., “m m m m m m m”). This is known as the changing-state effect (Jones and Macken, 1993). The changing-state effect does not arise when the task instructions are changed, so that serial recall (i.e., reproduction of the visual sequence with items reported back in the same order as they were presented) is no longer required. When, for example, the task is to identify the missing item from a closed set (e.g., the day of the week that was not presented), a changing-state sound sequence is no more disruptive than a steady-state sound sequence (Hughes, 2014). A leading explanation for this is that the changing-state effect only arises when the task requires serial rehearsal of the to-be-recalled items. Based on this explanation, it may be tempting to use the changing-state effect as an instrument to test whether another task, say mental arithmetic, involves serial rehearsal, by comparing the effects of a changing-state sound sequence with the effects of a steady-state sound sequence on mental arithmetic. If there is no difference between the two sound conditions, one might feel inclined to conclude that mental arithmetic does not require serial rehearsal. And similarly, if the changing-state effect arises, one might be tempted to conclude that mental arithmetic requires serial rehearsal. These conclusions/explanations are circular. They could be the best explanations, if they were the most probable, and they could even be correct, but they are still circular without some additional evidence for the assumption that the task—mental arithmetic—indeed involves (or does not involve) serial rehearsal.

Even if circular arguments can be acceptable when embedded into theories/explanations, the circularity that arises from confusing the definition of an effect with the explanation of that effect is never acceptable and should always be avoided. It would be wrong to say that the changing-state effect is an effect that takes place when the involuntary analysis of acoustic variability in a sound stream comes into conflict with the serial rehearsal of to-be-recalled items. Conversely, it would be right to say that the changing-state effect takes place when a difference is found between a changing-state sound condition and a steady-state sound condition, and the conflict between the processes is the explanation of why that difference arises.

## Summary and conclusion

The *definition* of an effect is the difference between two (or more) conditions whilst an *explanation* is a theory about why this difference arises. The definition and the explanation must not be confused, and the definition should never include the explanation. When the explanation of an effect is embedded into the definition of the effect, the interpretation of the effect is circular and conceptually weak. Some degrees of circularity may be acceptable, because the explanation may still be the most probable explanation, but not the circularity that is the result of confusing definition and explanation. The problems discussed in this paper can easily be avoided by always keeping in mind that the definition of an effect and the explanation of that effect are two different things.

### Conflict of interest statement

The author declares that the research was conducted in the absence of any commercial or financial relationships that could be construed as a potential conflict of interest.
